# Steps Toward a Universal Grammar of Dance: Local Grouping Structure in Basic Human Movement Perception

**DOI:** 10.3389/fpsyg.2019.01364

**Published:** 2019-06-18

**Authors:** Isabelle Charnavel

**Affiliations:** Department of Linguistics, Harvard University, Cambridge, MA, United States

**Keywords:** dance, movement, structure, grouping, perception, language, music

## Abstract

The general goal of this paper is to investigate the structure of our unconscious mental representation of dance: we do not perceive dance as an unanalyzed flow of movement, but we unconsciously create a mental representation regulated by structural principles. Specifically, this article examines local grouping principles in dance perception inspired by Lerdahl and Jackendoff's (1983) approach to musical grouping. I spell out the basic perceptual dimensions at work in basic human movement perception, and on that basis, I propose six principles of change that determine group boundaries in dance (change of body part, orientation, level, direction, speed, quality). I experimentally test the relevance and interaction of these principles, and find that they are organized on a scale of relative strength. This experiment thus supports the hypothesis that grouping is a general cognitive capacity applying across domains and modalities, and shows how specific grouping principles are stated in relation to modality-specific and domain-specific dimensions. More generally, it takes a step toward the development of a generative theory of dance that should help extend the research avenue of comparing complex temporal cognitive activities across modalities (visual, auditory) and purposes (referential, non-referential), which so far involves spoken language, signed language and music.

## Introduction

### Overarching Goal

When we watch people dancing, we do not perceive an unconstructed stream of movements, but we unconsciously turn this physical signal into a mentally produced organization regulated by specific principles. The overarching goal of this article is to investigate this type of structure as a way to further understand the organizational principles governing human cognition in general: exploring an understudied cognitive system—dance—from this perspective should shed new light on the underlying organization of our mental representations.

Language is one of the cognitive systems that has clearly been shown to be subject to a specific set of structural principles: the program of generative linguistics [developed by Chomsky in the late fifties (see Chomsky, 1981, i.a.)] largely consists in uncovering the relevant units and combination rules that determine the structure of language at different levels (e.g., at the level of sounds, words or sentences).

Moreover, a recent wealth of studies on sign languages (Brentari, 1998; Sandler and Lillo-Martin, 2006, among many others) has revealed that this type of principles is not modality-specific: despite their difference in modality (visual vs. auditory), sign and spoken languages are by and large part of the same abstract system.

Such structural principles are not domain-specific either: they are not restricted to referential systems like language that are mainly used to deliver messages about the external world, but they also apply in non-referential, artistic systems like music, as shown by Lerdahl and Jackendoff's (1983) pioneering work. Musical structure results from the interaction of several dimensions of organization such as grouping and meter (i.e., rhythm), as well as pitch, which have their own characteristic units and combinatorial principles [as summarized in Lerdahl and Jackendoff (1983)].

The goal of the present paper is to hypothesize that like music, dance is a non-referential cognitive system governed by structural principles: just as in the case of language, modality should not deeply affect the existence and the nature of organizational principles, so that musical structural principles should also be relevant in dance. This hypothesis challenges Patel's (2008: 306) claim that music does not have any counterpart in the visual modality: according to him, there is no “non-referential but richly organized system of visual signs with discrete elements and principles of syntax, created and shared for aesthetic ends by an appreciative community.”

Several studies have shown that human motion is subject to segmentation: identifying distinct acts within the dynamic motion flow is one basic component of action processing (Baldwin et al., 2008, i.a.). More specifically, event segmentation theory hypothesizes that the identification of different segments is largely tied to the actor's intentions (Zacks and Tversky, 2001, i.a.) and has investigated some neural correlates of event perception (Zacks et al., 2006, i.a.).

Brain activation related to event segmentation has also been revealed in dance-like actions (Schiffer and Schubotz, 2011; Pollick et al., 2012; Noble et al., 2014; Bachrach et al., 2016, i.a.). Furthermore, some studies based on artificial grammar learning show that humans can implicitly learn regularities in the structure of dance steps (Opacic et al., 2009, i.a.). Nevertheless, dance is usually not oriented toward a specific external goal, unlike most human actions: dance may be seen as serving an expressive purpose in conveying emotions (cf. expressionist dance, Rudolf von Laban), but it is not functional like everyday activities (e.g., doing the dishes); dance movements are usually seen as the intended outcome, not just as a means to an end (Schachner and Carey, 2013). Dance segmentation into sequences cannot therefore be based on the intended ultimate goal of the agent, but must rely on other factors (Bläsing, 2015).

The few recent psychology studies dealing with this issue mainly focus on factors related to the artistic aspect of dance: for instance, they show that dance segmentation is influenced by music, visual and motor expertise (Bläsing, 2015, i.a.), as well as aesthetic evaluation (Orgs et al., 2013, i.a.). Older studies in the field of dance ethnology, on the other hand, examine physical properties of movement to determine the structure of various types of dance so as to describe different dance traditions and their social and cultural significance (Kaeppler, 1972; Singer, 1974; Kürti, 1980; Bartenieff et al., 1984; Puri, 1986, i.a.).

The present article differs from both of these approaches: its general goal is neither to study the structure of dance as art, nor to describe the structural specificities of various dance traditions, but to examine the natural grammar of dance by determining the simplest and universal units of human movement and the way they are combined (cf. Napoli and Kraus, 2017; Patel-Grosz et al., 2018, for recent applications of linguistic methodology to dance analysis^1^).

Linguistic theory does not have the same object as literary and stylistic studies: linguists focus on everyday sentences to uncover the unconscious rules that govern their structure; they do not study the intentional rules (or their perception) of literary and poetic sentences related to art creation. Similarly, the goal here is not to study (the perception of) the structure of dance as created by a choreographer^2^, but the natural, unconscious and universal organizational principles of non-goal-oriented human movement. Just like in Lerdahl and Jackendoff's (1983) study of music, it is not the artistic or aesthetic aspect of dance that is of interest (see Calvo-Merino et al., 2008; Daprati et al., 2009; Christensen and Calvo-Merino, 2013, i.a.), but the grammar that underlies it and is implicitly manipulated to create artistically structured phenomena.

Furthermore, linguistic theory does not focus on grammars of particular languages for themselves, but aims to find universal and abstract linguistic properties that underlie the human linguistic faculty. Similarly, the general goal here is not to study structural specificities of particular dance styles, but to investigate fundamental and basic concepts of movement structure in order to shed light on a potential human capacity for dance perception. The specificities of dance (non-referentiality, no goal orientation, visual modality) should furthermore help us distinguish between modality-specific, domain-specific and general cognitive properties in the structure of unconscious mental representations.

### Perception of Grouping Structure

This article focuses on one type of organizational principle in dance: the grouping structure of the mental representation that observers construct when watching a dance performance^3^ [for an examination of the neural mechanisms in dance, see Sevdalis and Keller, 2011; Bläsing et al., 2012; Jola et al., 2013, i.a.]. Assuming that dance shares that type of structure with other cognitive systems, I aim to understand how grouping structure is realized in dance by taking the perspective of the perception [the motor component of dance will not be relevant here even if it has been shown to affect some aspects of observation since the discovery of the mirror neuron; see Calvo-Merino et al., 2006; Cross et al., 2006; Bläsing and Schack, 2012; Bläsing, 2015].

**Figure 1:**
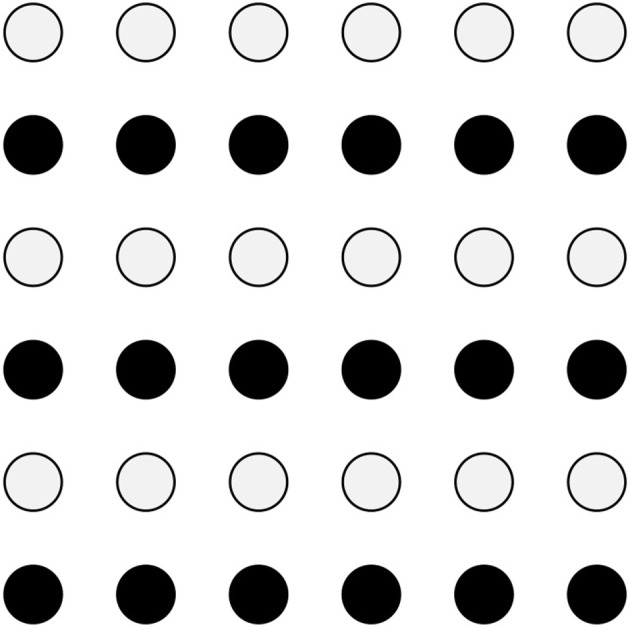
Gestalt law of similarity: visual perception. The white circles are perceived as forming groups distinct from the groups formed by the black circles.

Grouping principles have been proposed by psychologists of the Gestalt tradition in the domain of static visual perception (Wertheimer, 1938, i.a.). For instance, according to the law of similarity, stimuli that physically resemble each other tend to be perceived as grouping together (Figure 1); according to the law of proximity, objects that are close to each other tend to be perceived as forming a group. These principles have been shown to apply in other domains and modalities. In the domain of language, prosody is subject to grouping principles both in the auditory and in the visual modalities: in spoken languages, perceived prosodic grouping is mainly based on acoustic cues for the boundaries, such as duration, fundamental frequency and intensity (for reviews, see Shattuck-Hufnagel and Turk, 1996; Wagner and Watson, 2010, i.a.); in sign languages, prosody grouping is based on visual cues such as facial expression and movement of the body (Brentari, 1998, i.a.). In the domain of music, Lerdahl and Jackendoff (1983) demonstrate that the listener of a musical piece uses grouping principles to segment the raw sequences of notes that (s)he hears into hierarchical groups^4^.

Lerdahl and Jackendoff (1983) specifically propose that the grouping component in music consists of two sets of rules: well-formedness rules and preference rules^5^. Well-formedness rules state the conditions that all possible grouping structures must satisfy (i.e., a strict, non-overlapping, recursive hierarchy, where only contiguous sequences can constitute groups). Preference rules establish which of the formally possible structures that can be assigned to a piece correspond to the listener's actual intuitions. The need for preference rules follows from the nature of intuitive judgments involved in the case of music (preference judgments) as opposed to language (grammaticality judgments).

**Figure 2:**
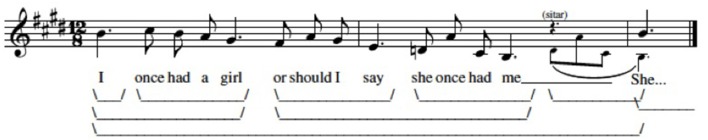
First phrase of Norwegian Wood with its grouping structure from Jackendoff and Lerdahl (2006 p. 38).

Grouping preference rules are of two types: local detail rules, which govern the perception of group boundaries, and more global rules that determine the organization of larger-level grouping. For instance, proximity (with respect to intervals of time in music) and similarity (with respect to register, dynamics, articulation and length in music) determine group boundaries, while symmetry and parallelism determine higher level grouping (Figure 2 shows an application of some of these grouping principles to an example). The relevance of such rules in the segmentation of the musical surface has been experimentally supported (Deliège, 1987, i.a.).

I hypothesize that the same kind of intuitive judgment is involved in the case of dance: the intuition of observers regards what is the most preferred way of understanding a choreography, that is, which representation is most coherent or natural; note that this question holds regardless of the observers' exposure to dance, even if the richness of the representations may vary accordingly. Therefore, I propose that similar well-formedness and preference rules of grouping can be transposed to dance.

### Local Detail Grouping Rules in Dance Perception

The experiment presented in this paper concentrates on one type of grouping preference rules in dance perception: the local rules that determine the boundaries between groups. This is based on the hypothesis that observers unconsciously rely on grouping rules to decide when a dance group ends and another one starts and thereby identify group units in the dance.

Specifically, I focus on one type of principle, the principle of similarity, which I hypothesize can determine grouping boundaries in dance^6^. In other words, dance is subject to local grouping rules of change.

**Figure 3:**
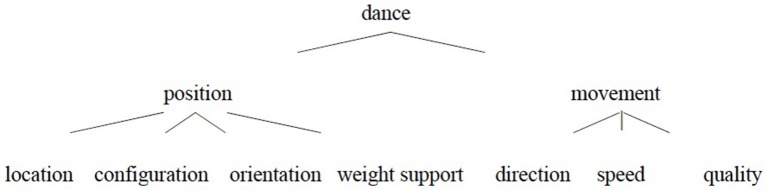
The hypothesized features of dance.

The specific physical properties of dance, I assume, determine what are the relevant parameters of change (cf. Napoli and Kraus, 2017). Sequences of the dancer's positions in space and time constitute the physical signal of dance (see Blake and Shiffrar, 2007, for a review of studies of the visual analysis of human motion by psychologists and cognitive neuroscientists). Dance is thus characterized both by the features of the dancer's position at a given time—location (of the dancer's body (parts) in space), configuration (of the body parts of the dancer), orientation [of the dancer's body (parts)], weight support—and by the features of the movement resulting from the sequences of positions—direction, speed, quality (see Figure 3). Note that most of these parameters are encoded in systems of dance notation such as Labanotation (von Laban, 1928) and that similar parameters are proposed for the phonology of sign languages (see Stokoe et al., 1976; Brentari, 1998, i.a.).

The first and main goal of my experiment is to test, on the basis of these dance features, the relevance of the following six local grouping rules of change in dance. Note that just as Jackendoff and Lerdahl (2006) treat a musical group as one of more adjacent notes in the musical surface, I consider a dance group as one or more adjacent positions in the dance movement; however, given the fundamental difference between music and dance with respect to continuity (musical notes are discrete, dance positions are continuous), group boundaries are not placed at transitions between two positions, but at positions themselves.

Consider a sequence of (non-contiguous) positions p_1_, p_2_, and p_3_.

1. **GPR 1 (change of moving entity)**: Position p_2_ may be seen as a group boundary if the set of body(s) (part(s)) affected by the change of positions p_1_-p_2_ is different from the set of body(s) (part(s)) affected by the change of positions p_2_-p_3_.
2. **GPR 2 (change of orientation)**: Position p_2_ may be seen as a group boundary if the orientation of the body (part) in p_1_-p_2_ is different from the orientation of the body (part) in p_2_-p_3_.
3. **GPR 3 (change of contact point with the floor/weight shift)**: Position p_2_ may be seen as a group boundary if the body part in contact with the floor in p_1_-p_2_ is different from the body part in contact with the floor in p_2_-p_3_.
4. **GPR 4 (change of direction)**: Position p_2_ may be seen as a group boundary if the path formed by p_1_-p_2_ does not have the same direction as the path formed by p_2_-p_3_.
5. **GPR 5 (change of speed)**: If p_2_-p_3_ forms a path of the same length as the path formed by p_1_-p_2_, position p_2_ may be seen as a group boundary if the amount of time taken to cover the path p_1_-p_2_ is different from the amount of time taken to cover the path p_2_-p_3_.
6. **GPR 6 (change of dynamics/quality)**: Position p_2_ may be seen as a group boundary if the quality of movement between positions p_1_-p_2_ is different from the quality of movement between positions p_2_-p_3_.

GPR1-GPR3 mainly concern the features of the dancer's position, and GPR4-GPR6 those of the movement itself.

GPR1 (change of moving entity) assumes that a group boundary is perceived each time a dancer joins or leaves the dance, or, at the level of a single dancer, each time a body part starts or stops moving. If we compare the grouping preference rules and Figure 3, it could seem that GPR1 corresponds to both the features “configuration” and “location.” In fact, I do not postulate any specific rule for location change because movement results from (any part of) the dancer changing location, so that location change cannot create grouping and is implied by the other rules (and the absence of location change, which could be relevant for grouping, amounts to immobility, which can be captured by GPR5, change of speed). GPR1 does not exactly amount to a change of configuration either: given that a human body is highly articulated (see kinematics and kinesiology for studies of such properties based on anatomy and geometry of motion), changes of configuration are highly complex and in part implied by other rules. GPR1 captures a specific type of configuration change (change of moving entity) that is perceptually relevant for grouping because it is neither implied by the existence of movement itself nor by the other types of change.

GPR2 (change of orientation) is based on the observation that a human body is intrinsically oriented (some body parts like hands are too), and implies that changes in orientation mark group transitions.

GPR3 (weight shift) takes into consideration the crucial role that weight plays in dance: as human movement is constrained by gravity, the part of the body that is in contact with the floor and supports the weight of the dancer is perceptually salient as a point of stability; this leads me to hypothesize that weight shifts are perceived as group boundaries. Note that weight shift is usually accompanied by other types of changes such as change of direction (GPR4) or change of moving body part (GPR1). But GPR3 is not redundant as it defines a grouping that extends until the contact point with the floor changes again.

GPR4 (change of direction) affects the two types of paths that can be perceived in dance: the path traced by the dancer in the performance area [cf. locomotion in Lasher (1981)], and the paths made in space by each body part (head, limbs …) moving independently of the whole body [cf. contour motion in Lasher (1981)]. The first type of movement (movement of the full body) is perceived as room-relative and happening in a quasi two-dimensional space due to the restriction imposed by gravity on the vertical plane, while the second type of movement (movement of the limbs) is perceived as body-relative and happening in a three-dimensional space due to the internal forces of the body compensating for gravity. GPR4 has to be distinguished from GPR2 because direction, a property of the movement, differs from orientation, a property of the moving entity; thus, a dancer can keep moving in the same direction (e.g., toward the audience) while changing orientation (e.g., facing the audience and then facing away from it), or conversely, a dancer can change direction (e.g., toward the audience and then toward the side of the scene) but not orientation (facing the audience the whole time).

GPR5 assumes that just like in music, changes of speed mark boundaries between groups (see Eitan and Granot, 2006 for an empirical investigation of how changes in musical parameters are associated with changes in movement).

Finally, GPR6 supposes that grouping is also determined by similarity in the quality or dynamics of the movement (see von Laban, 1966; Preston-Dunlop, 1987, and Laban Movement Analysis), which depends on the intensity of force (from gentle to strong) that is used and the way the energy is released (e.g., sudden, slow, sustained, jerky).

The prediction is that if these rules of change are relevant in the grouping perception of dance, observers watching human dance movements should tend to segment the movement based on these rules. If they are not relevant, observers should segment movements of dance without taking such changes into consideration. My experiment tests these predictions by examining simple arm movements.

### Conflicts in Local Detail Grouping Rules

The second, more exploratory goal of the experiment is to determine the relative strength of these local rules of change—if they indeed determine grouping—along the lines of Optimality Theory in phonology (see Prince and Smolensky, 1993, i.a.), in which a given rule x always overrides a rule y^7^.

**Figure 4:**
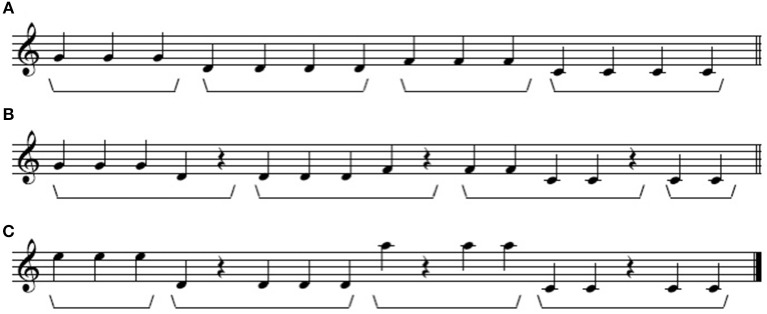
Conflicting grouping principles in music from Jackendoff and Lerdahl (2006: 39). **(A)** Grouping structure based on similarity of pitch. **(B)** Grouping structure based on temporal proximity. **(C)** Grouping structure based on similarity of pitch.

Lerdahl and Jackendoff (1983) argue that competition among conflicting principles is a normal feature in the determination of music structure because of the nature of judgments in music: to determine the grouping structure of musical events, listeners implicitly follow preference rules, which can be overriden in the case of interaction between rules. This contrasts with linguistic grammaticality judgment rules, which are absolute and cannot be violated. This is illustrated in Figure 4 with a toy example from Lerdahl and Jackendoff (1983 p. 39). Line (a) involves no conflict: the four groups are determined by similarity (in fact identity) of pitch. But the notes in lines (b) and (c) can be grouped in two different ways depending on whether we adopt similarity in pitch as a primary grouping principle (line c) or proximity in time (line b). Such conflicts have been experimentally tested by Deliège (1987).

**Figure 5:**
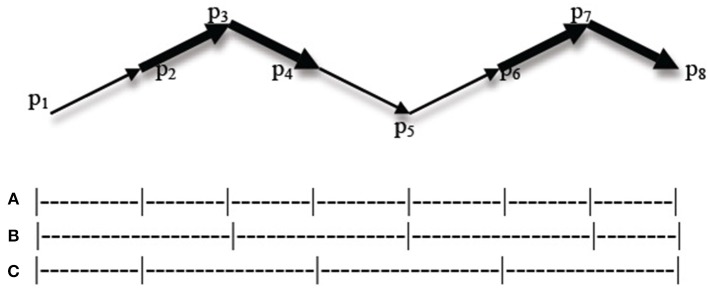
Conflicting grouping principles in dance (the contrast between bold and non-bold lines symbolizes speed changes). **(A)** Grouping structure based on both change of speed and change of direction. **(B)** Grouping structure based on change of direction. **(C)** Grouping structure based on change of speed.

The same kind of conflict should occur in dance, and the question addressed by the experiment is how such conflicts in dance grouping principles are resolved. Suppose that a change of direction and a change of speed occur at different times of the movement as illustrated in Figure 5 (direction changes in p_3_, p_5_, and p_7_, while speed changes in p_2_, p_4_, and p_6_).

There are several possibilities. One possibility is that both rules have the same strength and are cumulative, so that observers mark group boundaries at each type of change (line a). Another possibility is that one of the rules is stronger than the other one, so that observers always tend to perceive group boundaries for one type of change rather than the other one (e.g., direction as in line b, or speed as in line c). A further possibility is that rules do not vary in strength under normative conditions (e.g., direction and speed have the same strength as types of change), but they are used with different degrees of intensity (e.g., big change of direction vs. small change of speed); this presupposes that different types of change (e.g., direction vs. speed) can be compared to each other. This possibility could furthermore be combined with the second one: it may be the case that similarity rules under normative conditions vary in strength (e.g., direction change prevails over speed change in the general case), but their relative strength is affected by the relative intensity of change (e.g., a drastic change of speed prevails over a small change of direction). Finally, a last possibility is that the dance structure is simply perceived as ambiguous in such cases.

If we force observers to choose between line (b) and line (c), we predict that they will indifferently choose one or the other (i.e., the results should be at chance level) if the two rules have the same strength and the two changes have the same degree of intensity, or if the dance structure is ambiguous. However, we expect to obtain a significantly higher number of responses for one of the two choices if the two rules vary in strength under normative conditions or if they vary in intensity (whether or not they also vary in strength).

This means that if we control for the intensity of change, we can determine whether two local rules have different strengths. This is the second goal of the experiment in which I compare GPR1-GPR6 in pairs. Furthermore, if it turns out that local rules of change do have different strengths, a two-by-two comparison should allow us to establish a hierarchy of relative strength among these various rules.

## Experiment

The experiment addresses all these questions, summarized below, by forcing observers to choose between several segmentations of simple arm movements into two sequences:

- Are GPR1-GPR6 relevant for grouping perception in dance?
- If so, do GPR1-GPR6 have different strengths?
- If so, what is the relative strength of GPR1-GPR6?

I compare the six hypothesized local grouping rules GPR1-GPR6 two by two by constructing movements involving two types of changes happening at two different points of the movement and by asking observers to choose the most natural point of segmentation. The details of the experiment are presented below.

### Materials and Methods

#### Stimuli

The experiment included 15 target video clips (in format.mp4) ranging in length from 9 to 12 s at their actual filmed rate. Each clip featured the same female dancer performing a different arm movement with no accompanying music but a regular pulsation of 12–15 beats (tempo: 82 beats/min); the absence of music guaranteed that participants could not be based on musical structure to segment the movement (see Bläsing, 2015, about the effects of music on dance segmentation). In each case, the movement started on beat 4 or 5 and ended on beat 11 or 12, respectively: some time of immobility always preceded and followed the movement. Macintosh iMovie software was used to incorporate a visual timer on the video (i.e., the number 1, 2, … etc., appeared on top of the video while the first, second, … etc, beat was heard).

The 15 movements crucially differed in the types of changes affecting them. In each case, the continuous movement underwent two changes at two different points: a first type of change (among the changes described in GPR1-GPR6) occurred when around one third of the movement was performed, and a second, different type of change occurred when around two thirds of the movement were executed. The same amount of time thus elapsed between the beginning of the movement and the first change, between the two changes, and between the second change and the end of the movement. This was meant to guarantee that temporal proximity could not confound the relative strength of the similarity rules. Moreover, the changes did not always occur at the same beats (e.g., 6 and 9) to prevent participants from adopting a unique strategy across the experiment (e.g., always choose beat 6).

Given that one of the goals of the experiment was to compare the relative strengths of the six similarity rules, it was also important to control for the saliency and intensity of each change (as discussed in section Conflicts in Local Detail Grouping Rules), which depends on the particulars of each variable. This required establishing a normative state for each condition. To keep the experiment simple and therefore more easily interpretable, I chose changes that only affected arms for rules GPR1-2,4-6. Arm movements present several advantages: they are subject to all types of change (except weight shift) without affecting other body parts and independently of weight shift (as opposed to leg movements, in particular), and they only concern one type of path (contour motion vs. locomotion, see Lasher, 1981, and discussion above about GPR4). This meant that I chose an intermediate level of saliency for each condition: arm movements are less salient than leg movements, but more so than finger movements, for instance. Accordingly, I chose to realize GPR3 (change of contact point with the floor/weight shift) as a level change (i.e., rising on both tiptoes or conversely lowering both heels), which is not as salient as a weight shift from one foot to the other, for instance, but is more salient that a level change only affecting one part of the body (e.g., one foot or one hand). Level change was furthermore appropriate for the experiment as a test for weight shift because it does not imply any other type of change (body part, direction or orientation, in particular) and can thus be examined independently.

This intermediate level of saliency in the particulars of each variable was combined with a high degree of change intensity. This choice was guided by two facts: first, GPR1 (change of moving entity) had to be tested using a categorical change (change of right vs. left arm) given the nature of the rule, and a categorical change is perceived as intense; second, this was meant to compensate the intermediate level of saliency in the particulars of each variable and thus make the conditions of the experiment sufficiently perceptible. The condition for GPR2 (change of orientation) involved an orientation change of 180° of the moving arm, which is the most drastic change of orientation (at the level of the dancer, this amounts to facing the audience and then facing away from the audience)^8^. As mentioned above, GPR3 was tested with a level change from tiptoes to flat feet or vice versa, which is the maximal degree of level change possible for (nude) feet. The condition for GPR4 (change of direction) consisted of a direction change of 90° of the arm movement: I consider a direction change of 90° to be perceived as the most drastic one because this angle corresponds to the biggest angle between two distinct paths (on the contrary, the path remains the same in the case of a direction change of 180°) and is not used in natural movements. The condition for GPR5 (change of speed) involved an alternation of slow and quick movements of the arm, which was performed as intensely as possible under the indicated constraints of space and time. Finally, the condition for GPR6 (change of dynamics/quality) was also tested using two highly contrastive qualities of movement, namely smooth vs. jerky movements (of the moving arm).

These 6 possible types of hypothesized changes (GPR1-GPR6) yielded 15 pairs of different changes presented in Table 1. Assuming that participants cannot remain fully concentrated for more than 30 min, I did not test all possible orders to keep the length of the experiment reasonable. But to make sure that the relative order of change occurrences did not interfere, each possible type of change (e.g., direction) occurred first in 2–3 items and second in the remaining 2–3 items involving it (given that 6 types of changes were tested by using combinations of two different types of changes, each type occurred in 5 items) as shown in Table 1.

**Table 1:** The combinations of 2 possible changes in the 15 stimuli of the experiment.

	**First change** **(at 1/3 of the movement)**	**Second change** **(at 2/3 of the movement)**
Item 1	Body part (GPR1)	Orientation (GPR2)
Item 2	Level (GPR3)	Body part (GPR1)
Item 3	Direction (GPR4)	Body part (GPR1)
Item 4	Body part (GPR1)	Speed (GPR5)
Item 5	Body part (GPR1)	Quality (GPR6)
Item 6	Orientation (GPR2)	Level (GPR3)
Item 7	Orientation (GPR2)	Direction (GPR4)
Item 8	Orientation (GPR2)	Speed (GPR5)
Item 9	Quality (GPR6)	Orientation (GPR2)
Item 10	Direction (GPR4)	Level (GPR3)
Item 11	Level (GPR3)	Speed (GPR5)
Item 12	Level (GPR3)	Quality (GPR6)
Item 13	Direction (GPR4)	Speed (GPR5)
Item 14	Direction (GPR4)	Quality (GPR6)
Item 15	Quality (GPR6)	Speed (GPR5)

The 15 movements also differed in other irrelevant respects because of physical constraints or for making the task more entertaining and natural to participants. For instance, the gaze of the dancer followed the movement of her arm. Table 2 includes still images showing the crucial points of each movement. The 15 full video clips are included in the Supplemental Files.

**Table 2:** Illustrative still-images extracted at 4 crucial points from each video clip utilized in the experiment (the depicted individual provided a written informed consent for publication of these images).

	**Starting position** **(between beat 1 and beat 4–5)**	**First change** **(occurring at beat 6–7)**	**Second change** **(occurring at beat 9–10)**	**Finishing position** **(between beat 9–10 and beat 12–15)**
Item 1 (body part/orientation)	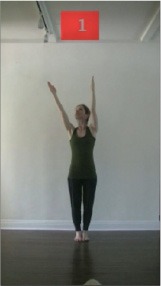	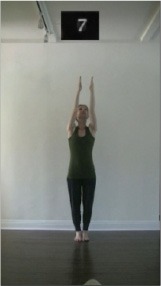	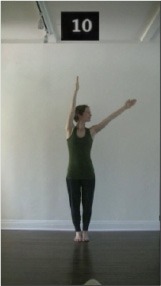	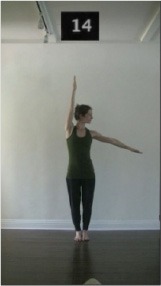
Item 2 (level/body part)	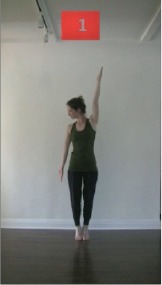	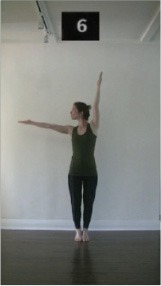	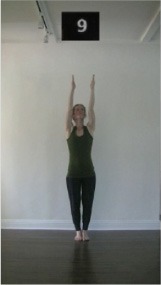	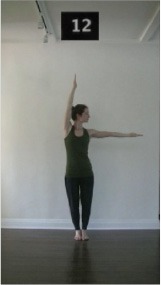
Item 3 (direction/body part)	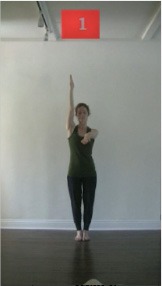	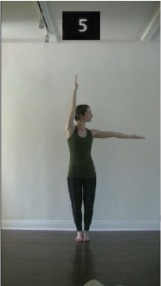	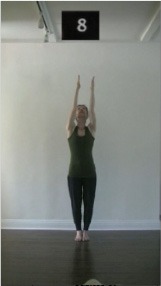	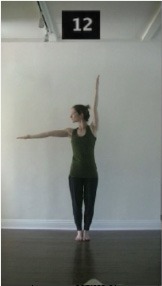
Item 4 (body part/speed)	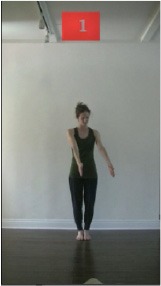	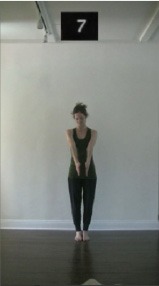	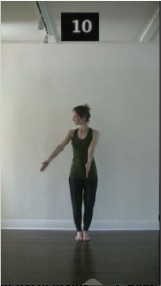	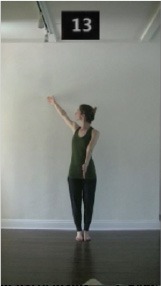
Item 5 (body part/quality)	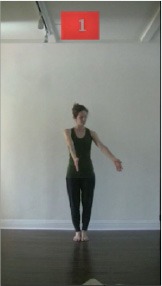	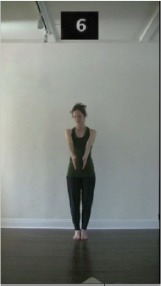	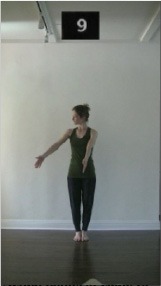	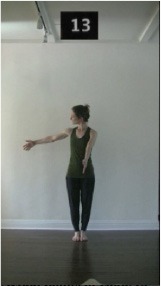
Item 6 (orientation/level)	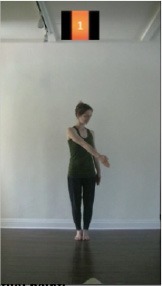	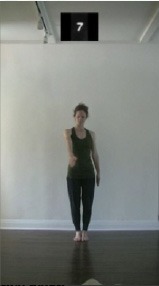	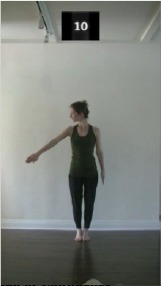	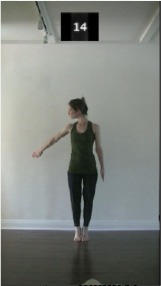
Item 7 (orientation/direction)	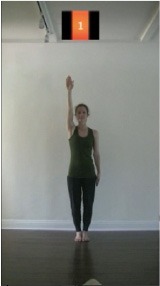	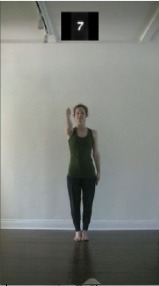	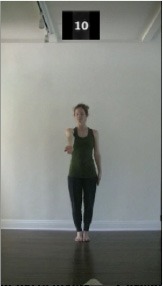	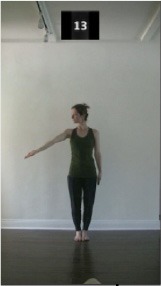
Item 8 (orientation/speed)	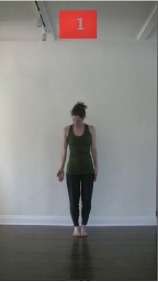	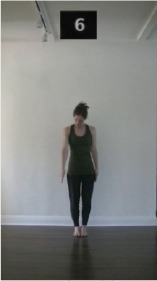	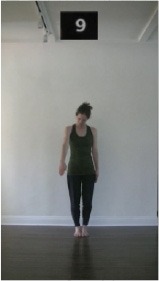	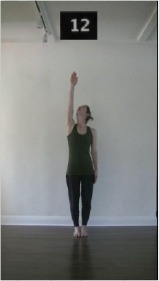
Item 9 (quality/orientation)	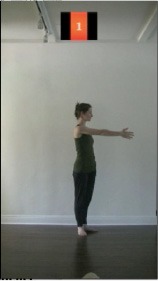	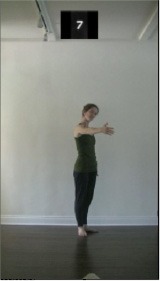	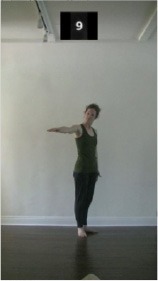	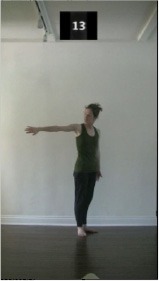
Item 10 (direction/level)	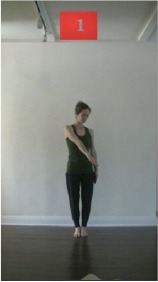	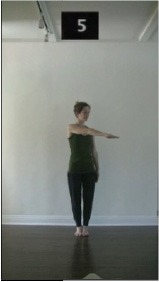	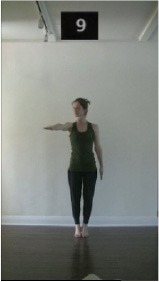	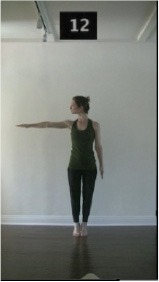
Item 11 (level/speed)	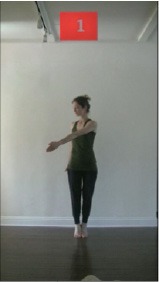	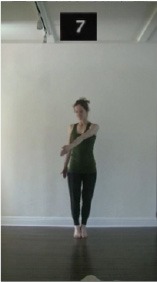	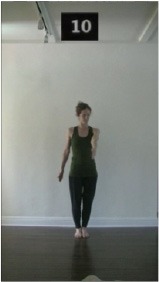	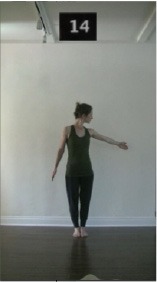
Item 12 (level/quality)	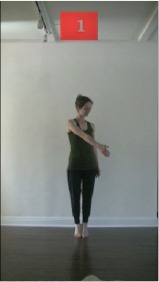	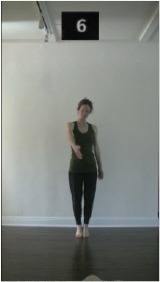	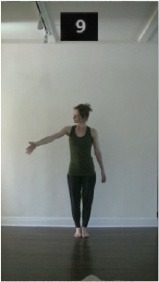	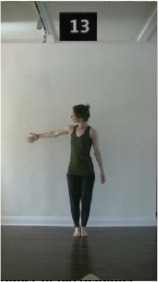
Item 13 (direction/speed)	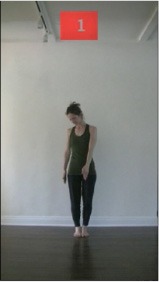	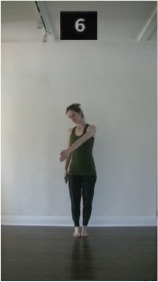	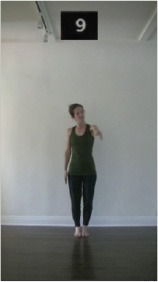	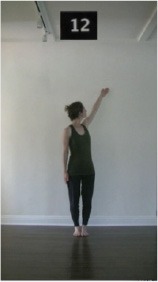
Item 14 (direction/quality)	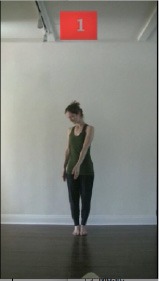	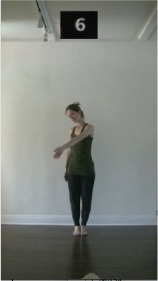	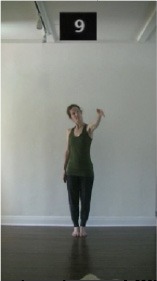	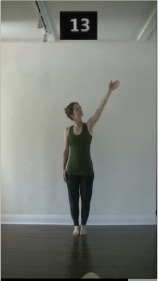
Item 15 (quality/speed)	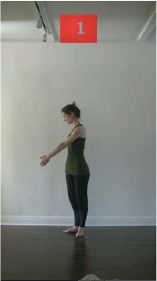	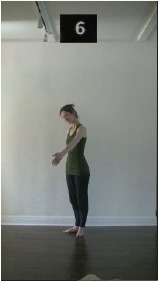	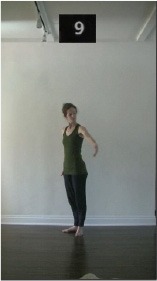	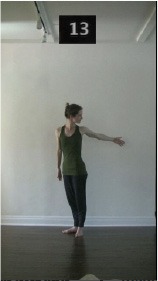

#### Participants

Fifty participants took part in the experiment over the internet, via the Amazon Mechanical Turk website (MTurk, https://www.mturk.com), which automatically directed them to the survey run on Qualtrics. To participate, individuals were required to have had at least 97% of their previous work judged as acceptable. All participants gave informed consent,^9^ and they were paid $1 for approximately 15 min of their time (median of total duration of experiment: 14′′18′). Repeat participation by the same individual was not allowed (each participant was identified by a unique and stable code by the MTurk system).

Among these 50 participants, 20 participants were excluded for failing more than one of the 8 attention checks described in the procedure section (17), for having already taken the survey (1), or for indicating clear misunderstanding of the instructions (2).

All 30 remaining participants^10^ had the same level of dance: none of them was a regular dancer or choreographer, but all of them reported having had some exposure to dance either by taking occasional dance classes or by occasionally watching dance performances (see Bläsing and Schack, 2012; Bläsing (2015), i.a., for effects of dance expertise on segmentation).

#### Procedure

After the consent page, participants read instructions and familiarized themselves with the survey by playing with a sample video clip (it was possible for them to play each video as many times as they wanted and to pause it at any time). They were instructed to choose between several possible segmentations of the movement based on their intuition. Specifically, after watching each of the videos, they were asked the following question: “When is it most natural to cut the movement into two sequences?” and they were presented with one of the two sets of answers described in Table 3. Note that I used the expression “around” for two reasons: all changes did not happen in an instant (e.g., the orientation change of the hand takes almost 1 s), and a given number remained on the screen for 730 ms. If a change of speed occurs 500 ms after the number 6 appears, it is therefore not clear whether participants perceiving a boundary there should choose 6 (the speed change indeed occurred while 6 was on the screen) or 7 (the speed change occurred closer to the change from 6 to 7 than to the change from 5 to 6).

**Table 3:** Question and answer sets of the experiment randomized across stimuli.

**Target question**	**When is it most natural to cut the movement into two sequences?**						
Answer set 1	Around 4	Around 5	Around 6	Around 7	Around 8	Around 9	Around 10
Answer set 2	Around 5	Around 6	Around 7	Around 8	Around 9	Around 10	Around 11

**Figure 6:**
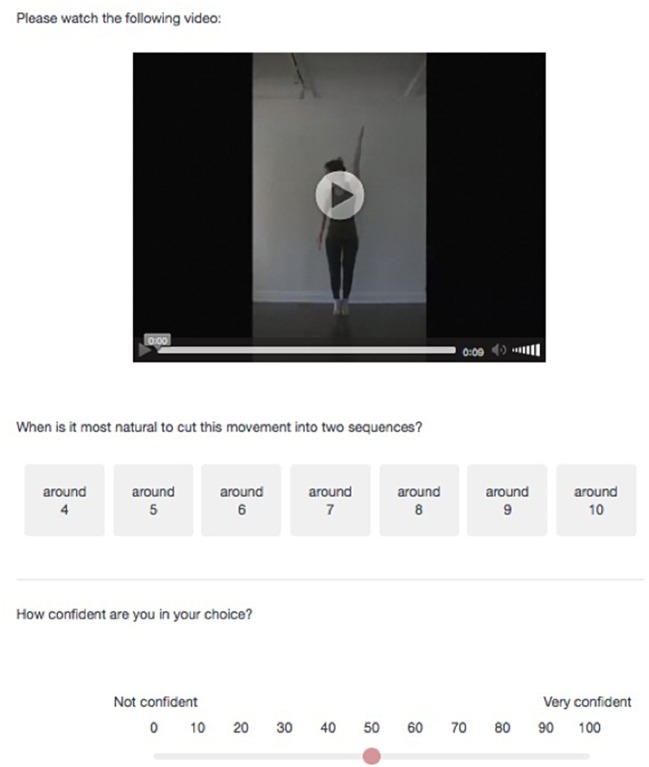
Sample of question item (the depicted individual provided a written informed consent for publication of this image).

On the same page, they were then asked to indicate how confident they are in their choice by dragging the slider to the appropriate point between 0 and 100%. Participants had to provide an answer to each question to move forward, and could not return to previous pages after moving forward to a new page. A sample question is shown in Figure 6.

The order of questions was randomized, and 8 attention checks were included at 8 random points of the experiment. These consisted in different multiple-choice questions aiming to check that the videos had been carefully watched, such as “In the previous video, how many arms did the dancer move?” or “In the previous video, what was the dancer looking at?” At the end of the experiment, participants were asked about their level of exposure to dance and were given the opportunity to provide comments.

### Predictions

Recall that the experiment had 3 goals:

- Are GPR1-GPR6 relevant for grouping perception in dance?
- If so, do GPR1-GPR6 have different strengths?
- If so, what is the relative strength of GPR1-GPR6?

Regarding question (i), the prediction is that if GPR1-GPR6 are relevant for grouping in dance, participants should systematically choose to cut the movement at one of the two points corresponding to the two types of changes; they should not select intermediate beats as points of segmentation. On the contrary, if these rules are not relevant, participants should randomly choose among the 7 proposed beats.

**Figure 7:**
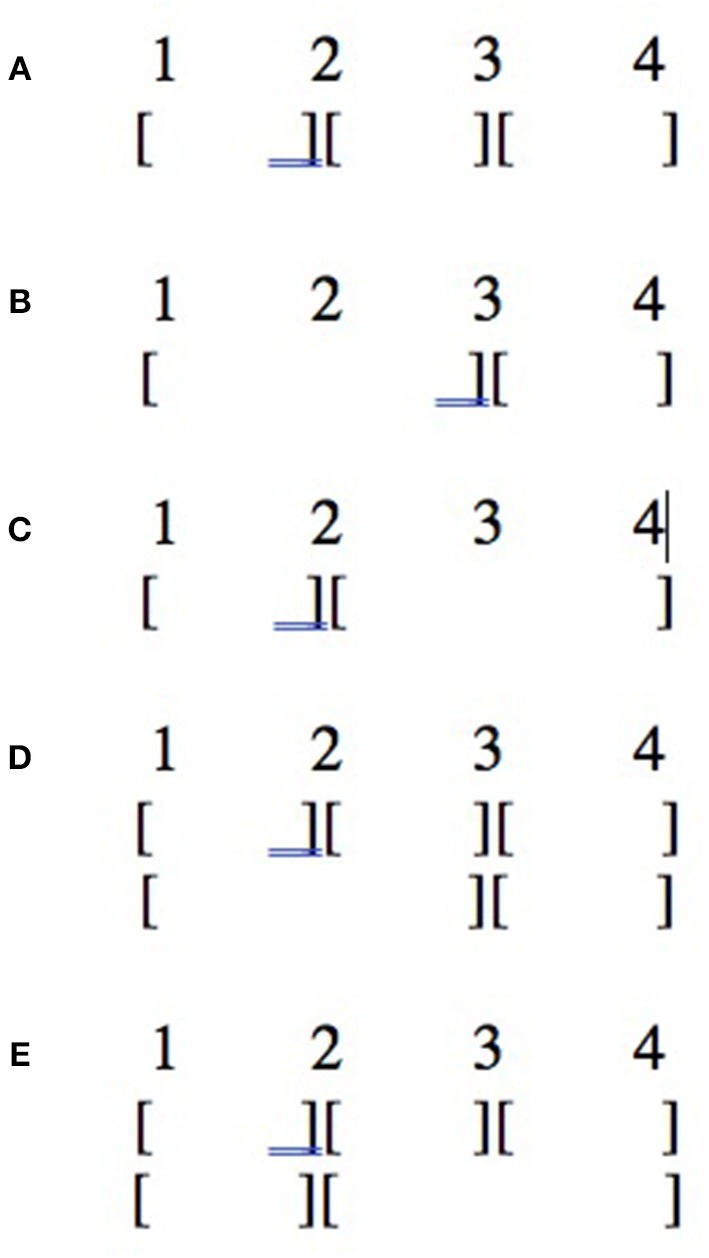
Several possible segmentations of the movement into two sequences. 1, 2, 3, and 4, respectively, correspond to the starting position, the point at which the first type of change occurs, the point at which the second type of change occurs, and the finishing position. **(A)** One-level grouping structure based on both types of change. **(B)** One-level grouping structure based on the second type of change. **(C)** One-level grouping structure based on the first type of change. **(D)** Two-level grouping structure based on both types of change at the lower level of representation and on the second type of change at the higher level of representation. **(E)** Two-level grouping structure based on both types of change at the lower level of representation and on the first type of change at the higher level of representation.

Question (ii) is only relevant under the first option, i.e., if GPR1-GPR6 are used for the segmentation of dance movements. In that case, the prediction is that if GPR1-GPR6 have the same strength, participants should randomly choose among the two possible types of changes to cut the movement into two sequences. Otherwise, they should tend to select the point corresponding to the stronger type of change. More specifically, the first scenario (random choice between the two types of changes) is expected to happen if observers apply both rules indiscriminately as in line (a) of Figure 7 below, or if they randomly choose between each rule to apply (i.e., they randomly choose between line b and line c); both can also happen simultaneously (i.e., they randomly choose between line d and line e), if we suppose that there are two levels of grouping, and that each rule applies at the lower level, but only the stronger rule applies at the higher level. Conversely, the second scenario (selection of the stronger type of change) is predicted to happen if all observers tend to select one of the two structures in line (b) and line (c), or in line (d), and line (e).

Finally, question (iii) is only relevant if the second scenario occurs in more than one case, i.e., if several grouping preference rules differ in strength. In that case, the prediction is that by comparing the 15 two-by-two comparisons, we should be able to establish a hierarchy of the 6 grouping rules. In particular, the two-by-two comparisons are expected to be compatible with each other based on the assumption that transitivity between the rules should hold.

### Results

The results are presented in Table 4. For each item, the first line indicates when exactly the two types of change occurred in the stimuli (i.e., when the blue dotted lines appear): for instance, the first change of item 1 (change of body part) happened when beat 6 disappeared and beat 7 appeared on the screen, and the second change (change of orientation) occurred almost during the whole duration the number 10 was on the screen. The second line includes the number of participants (out of 30) that selected each answer: for instance, 19 participants chose the answer “around 7” for the first item; “n/a” means that the corresponding number was not proposed as a possible answer (recall that the same numbers were not proposed in each case to avoid repetitive strategies, see Table 3 above). Finally, the third line shows the percentage of participants that selected each answer: for instance, 63.3% of participants chose the answer “around 7” for the first item. Furthermore, note that results about confidence rates for each type of answer can be found in the Supplementary Files; these additional results are however only suggestive given the significantly different number of participants in each case.

**Table 4:** Detailed results by item: number and percentage of participants for each answer option.

		**Answer options (beat number appearing on the screen)**							
	**Item**	**≈4**	**≈5**	**≈6**	**≈7**	**≈8**	**≈9**	**≈10**	**≈11**
Actual points of change	Item 1-body part/orientation			-	-			---------	
Number of participants		n/a	0	7	19	2	0	2	0
Percentage of participants		n/a	0	23.33	63.33	6.67	0	6.67	0
Actual points of change	Item 2-level/body part				-------		--		
Number of participants		1	0	4	1	11	13	0	n/a
Percentage of participants		3.33	0	13.33	3.33	36.67	43.33	0	n/a
Actual points of change	Item 3-direction/body part		--				--		
Number of participants		1	6	0	0	12	11	0	n/a
Percentage of participants		3.33	20	0	0	40	36.67	0	n/a
Actual points of change	Item 4-body part/speed				--			---	
Number of participants		n/a	0	2	20	0	3	5	0
Percentage of participants		n/a	0	6.67	66.67	0	10	16.67	0
Actual points of changes	Item 5-body part/quality			--			---		
Number of participants		0	2	26	2	0	0	0	n/a
Percentage of participants		0	6.67	86.67	6.67	0	0	0	n/a
Actual points of change	Item 6-orientation/level				--------			-------	
Number of participants		n/a	1	1	11	0	3	14	0
Percentage of participants		n/a	3.33	3.33	36.67	0	10	46.67	0
Actual points of change	Item 7-orientation/direction					--------		--	
Number of participants		n/a	1	1	4	6	2	15	1
Percentage of participants		n/a	3.333	3.333	13.33	20	6.67	50	3.33
Actual points of change	Item 8-orientation/speed				--------		---		
Number of participants		0	0	2	11	9	7	1	n/a
Percentage of participants		0	0	6.67	36.67	30	23.33	3.33	n/a
Actual points of change	Item 9-quality/orientation				---		---------		
Number of participants		0	0	0	0	4	23	3	n/a
Percentage of participants		0	0	0	0	13.33	76.67	10	n/a
Actual points of change	Item 10-direction/level			--			-------		
Number of participants		1	7	5	1	4	12	0	n/a
Percentage of participants		3.33	23.33	16.67	3.33	13.33	40	0	n/a
Actual points of change	Item 11-level/speed				----------			---	
Number of participants		n/a	0	2	14	2	5	7	0
Percentage of participants		n/a	0	6.67	46.67	6.67	16.67	23.33	0
Actual points of change	Item 12-level/quality				---		---		
Number of participants		0	1	19	9	1	0	0	n/a
Percentage of participants		0	3.33	63.33	30	3.33	0	0	n/a
Actual points of change	Item 13-direction/speed				---		---		
Number of participants		0	0	12	3	4	10	1	n/a
Percentage of participants		0	0	40	10	13.33	33.33	3.33	n/a
Actual points of change	Item 14-direction/quality			--			---		
Number of participants		3	4	20	1	0	2	0	n/a
Percentage of participants		10	13.33	66.67	3.33	0	6.67	0	n/a
Actual points of change	Item 15-quality/speed			---			---		
Number of participants		3	0	1	5	7	13	1	n/a
Percentage of participants		10	0	3.33	16.67	23.33	43.33	3.33	n/a

#### Question (i): Relevance of Grouping Rules of Change

Based on these results, we can answer question (i) by the positive: participants systematically chose points at which changes happened to cut the movement into two sequences. This is made obvious in Table 5, where for each item, it is indicated how many (percents) of participants selected a target point, a near-target point and a non-target point. By target point, I mean that the beat number selected as the answer is the number that was on the screen when the change happened. By near-target, I mean that the beat number selected as the answer appeared <500 ms before or after the target number. Non-target points are the remaining numbers that meet neither of these conditions. Note that merging target and near-target points would be justifiable as it is reasonable to suppose that choosing near-target points amounts to using move changes as criteria of segmentation. For instance, the change of body part in the item “direction/body part” occurred while the number 8 was on the screen (and 12 participants chose this target point), but just before the number 9 appeared; the most plausible hypothesis is that the 11 participants who chose the near-target number 9 chose that number because they perceived the change of body part as occurring around the number 9. But as a precaution, I analyzed the results in two ways, first by counting near-target answers as target answers, and then by counting near-target answers as non-target answers. As explained below, the results remain the same in both cases, which confirms their validity. Both binomial tests and paired two-tailed *t*-tests show that both the difference between target and near-target answers on the one hand and non-target answers on the other hand, and the difference between target answers on the one hand and near- and non-target answers on the other hand, are significant. The binomial tests (computed in the last two columns of Table 5 for each item and for all items) test the statistical significance of the deviation from the distribution expected under chance conditions into the two relevant categories (target vs. near-and non-target answers in the first column; target and near-target vs. non-target answers in the second column). Under chance conditions, the participants have a probability of 2/7 to choose target answers, given that among the 7 proposed answers, 2 correspond to target points (the probability is 3/7 in the few conditions where 2 beat numbers appear on the screen while one of changes is happening as shown in Table 4 above; this is taken into consideration in the calculation), and they have a probability of 4/7 to choose target/near-target answers (or 3/7 for some items). For each item and overall, there is significant deviation from this distribution expected under chance conditions (*p* < 0.001 for most cases and *p* < 0.05 for all cases). This means that participants non-randomly choose to segment the movement at points of change; only a negligible number of participants (an average of 5.11% across all items) cut the movement at non-target points.

**Table 5:** For each item, number and percentage of participants who chose target points, near-target points and non-target points, and corresponding binomial tests.

**Item**		**Target**	**Near-target**	**Non-target**	**Binomial test (target vs. near- and non-target)**	**Binomial test (target and near-target vs. non-target)**
Body part/orientation	Number of participants	28	0	2	*p* < 0.001	*p* < 0.001
	Percentage of participants	93.33	0	6.67		
Level/body part	Number of participants	28	1	1	*p* < 0.001	*p* < 0.001
	Percentage of participants	93.33	3.33	3.33		
Direction/body part	Number of participants	18	12	0	*p* < 0.001	*p* < 0.001
	Percentage of participants	60	40	0		
Body part/speed	Number of participants	28	2	0	*p* < 0.001	*p* < 0.001
	Percentage of participants	93.33	6.67	0		
Body part/quality	Number of participants	26	2	2	*p* < 0.001	*p* < 0.001
	Percentage of participants	86.67	6.67	6.67		
Orientation/level	Number of participants	25	4	1	*p* < 0.001	*p* < 0.001
	Percentage of participants	83.33	13.33	3.33		
Orientation/direction	Number of participants	25	2	3	*p* < 0.001	*p* < 0.001
	Percentage of participants	83.33	6.67	10		
Orientation/speed	Number of participants	20	9	1	*p* = 0.005	*p* < 0.001
	Percentage of participants	66.67	30	3.33		
Quality/orientation	Number of participants	23	7	0	*p* < 0.001	*p* < 0.001
	Percentage of participants	76.67	23.33	0		
Direction/level	Number of participants	19	9	2	*p* < 0.001	*p* < 0.001
	Percentage of participants	63.33	30	6.67		
Level/speed	Number of participants	23	5	2	*p* < 0.001	*p* < 0.001
	Percentage of participants	76.67	16.67	6.67		
Level/quality	Number of participants	19	11	0	*p* < 0.001	*p* < 0.001
	Percentage of participants	63.33	36.67	0		
Direction/speed	Number of participants	22	7	1	*p* < 0.001	*p* < 0.001
	Percentage of participants	73.33	23.33	3.33		
Direction/quality	Number of participants	22	4	4	*p* < 0.001	*p* < 0.001
	Percentage of participants	73.33	13.33	13.33		
Quality/speed	Number of participants	14	12	4	*p* = 0.016	*p* < 0.001
	Percentage of participants	46.67	40	13.33		
Overall	Number of participants	340	87	23	*p* < 0.001	*p* < 0.001
	Percentage of participants	75.56	19.33	5.11		

Binomial tests suppose that the trials are independent. For each item, the answers given by the different participants are indeed independent since they cannot see each other's answers. But given that each participant answers several questions (one per condition), it might not be ideal to consider all trials as independent: the first answers one participant provides could influence her next answers. To make sure that this does not affect the significance of the results, I also performed paired two-tailed *t*-tests. First, I grouped target and near-target answers together and compared their means with those of non-target answers: they are significantly different whether the answers are averaged by item (*p* < 0.001) or by participant (*p* < 0.001). Second, I compared the averages of target answers with those of near- and non-target answers, and this difference also comes up as significant whether it is calculated by item (*p* < 0.001) or by participant (*p* < 0.001).

In sum, these results strongly support the first hypothesis that GPR1-GPR6 are used for movement segmentation.

#### Questions (ii) and (iii): The Relative Strength of GPR1-GPR6

**Figure 8:**
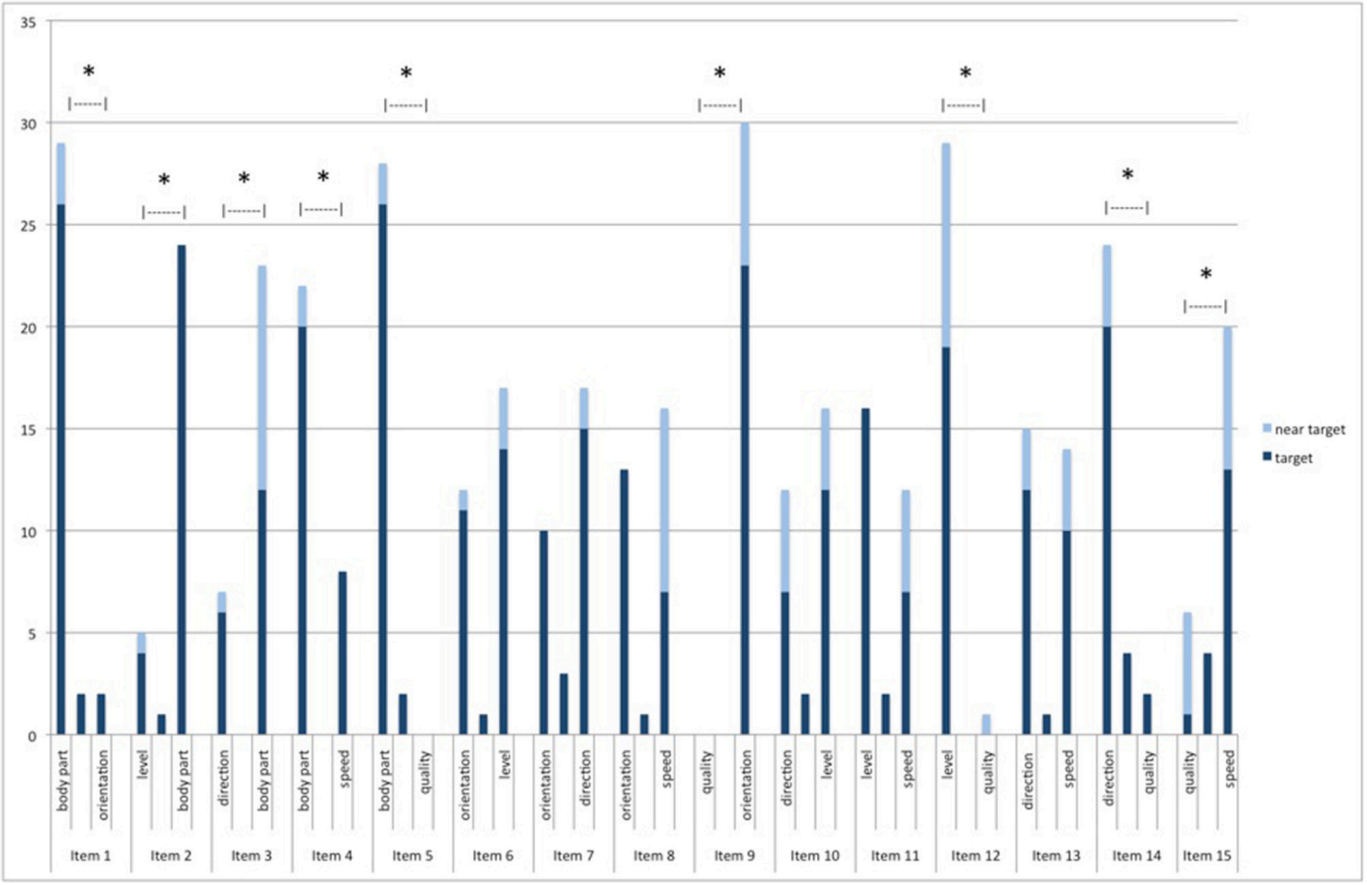
Number of participants selecting each type of change as a point of segmentation—For each item, the first column indicates the number of participants who chose the (near-)target point corresponding to the first type of change (e.g., change of body part for item 1), the third column indicates the number of participants who chose the (near-)target point corresponding to the second type of change (e.g., change of orientation for item 1), and the middle column indicates the number of participants who chose non-target points. *Indicates statistical significance.

The results also support the second hypothesis according to which not all rules GPR1-GPR6 have the same strength, as made clear by Table 6 and Figure 8 below.

**Table 6:** Relative strength of the six types of changes: for each change in each item, number of participants who chose target, near-target points and both, binomial tests and ranking of each pair of changes.

**Item**		**Target**	**Near-target**	**Target +near-target**	**Binomial test**	**Relative strength**
1	Body part	26	0	26	*p* < 0.001	^*^Body part > orientation
	Orientation	2	0	2		
2	Level	4	1	5	*p* = 0.029	^*^Body part > level
	Body part	24	0	24		
3	Direction	6	1	7	*p* = 0.002	^*^Body part > direction
	Body part	12	11	23		
4	Body part	20	2	22	*p* < 0.001	^*^Body part > speed
	Speed	8	0	8		
5	Body part	26	2	28	*p* < 0.001	^*^Body part > quality
	Quality	0	0	0		
6	Orientation	11	1	12	*p* = 0.097	Level > orientation
	Level	14	3	17		
7	Orientation	10	0	10	*p* = 0.063	Direction > orientation
	Direction	15	2	17		
8	Orientation	13	0	13	*p* = 0.126	Speed > orientation
	Speed	7	9	16		
9	Quality	0	0	0	*p* < 0.001	^*^Orientation > quality
	Orientation	23	7	30		
10	Direction	7	5	12	*p* = 0.113	Level > direction
	Level	12	4	16		
11	Level	16	0	16	*p* = 0.113	Level > speed
	Speed	7	5	12		
12	Level	19	10	29	*p* = 0.002	^*^Level > quality
	Quality	0	1	1		
13	Direction	12	3	15	*p* = 0.144	Direction > speed
	Speed	10	4	14		
14	Direction	20	4	24	*p* = 0.002	^*^Direction > quality
	Quality	2	0	2		
15	Quality	1	5	6	*p* = 0.003	^*^Speed > quality
	Speed	13	7	20		

* *Indicates statistical significance*.

Binomial *t*-tests (indicated in the next to last column of Table 6) show that the choice between the two types of change as the point of segmentation (target and near-target points) significantly differs from chance level in most cases (*p* < 0.05 for items 1, 3, 4, 5, 8, 9, 10, 14, 15): participants non-randomly chose one type of change over the other one in those cases (marked with an asterisk in the last column). For instance, 26 participants chose the first change of item 1 (i.e., change of body part) as the point of segmentation, but only 2 chose the second one (i.e., change of orientation). Note that unlike in the previous case, binomial tests can be safely used here as each participant only saw each type of pair of changes once: all trials (i.e., all answers of different participants) are independent^11^.

Furthermore, the order of appearance did not interfere in the results: a heteroscedastic *t*-test shows that changes (target and near-target points) appearing first were not significantly more or less selected than changes appearing second, whether the *t*-test was performed between items (*p* = 0.65) or participants (*p* = 0.17).

These two-by-two comparisons allow us to establish a scale for our six hypothesized rules of change. The two extremes are clear, as shown in (7): change of body part (GPR1) significantly prevails over all other changes, and change of quality (GPR6) is significantly weaker than all other changes. Note that for that reason, it remains unclear whether quality change can be a point of segmentation: since other changes are stronger, very few participants choose to cut the movement when the quality of the movement changes.

**7. Strength scale of grouping preference rules** (based on statistical significance): body part (GPR1) > level (GPR3)/direction (GPR4)/speed (GPR5)/orientation (GPR2) > quality (GPR6).

The differences between the 4 other types of changes are more subtle (they are not statistically significant, but there is a clear tendency). Based on the number values, we observe that their relative ordering gives rise to a coherent scale shown in (8): the two-by-two comparisons never contradict each other, but respect transitivity, which supports the validity of the scale.

**8. Strength scale of grouping preference rules** (based on number values) body part: (GPR1) > level (GPR3) > direction (GPR4) > speed (GPR5) > orientation (GPR2) > quality (GPR6).

### Discussion

The main and most robust result of the experiment is to support the hypothesis that dance obeys grouping principles, just like other cognitive domains such as language (esp. prosody), music or vision. The segmentation relies on general and implicit structural principles, which can be stated in relation to visual parameters specific to human movement.

Specifically, the experiment has confirmed that configuration (esp. which body part is moving), weight (esp. which part of the body supports the weight of the dancer), and orientation of the dancer (body parts), as well as direction, speed, and (potentially) quality of the movement are properties that observers take into account to cut human movement into sequences. These properties specific to the visual modality and to temporal activities are used for movement segmentation based on the general principle of similarity: one sequence of movement can be characterized as a group unit as long as these parameters remain unchanged; in other words, a change in one of these parameters marks a group boundary, as specified by the grouping preference rules GPR1 (change of moving entity), GPR2 (change of orientation), GPR3 (change of contact point with the floor/weight shift), GPR4 (change of direction), GPR5 (change of speed), and GPR6 (change of dynamics/quality) defined above in (1-6).

Moreover, the experiment suggests that these rules can be organized into a scale of strength shown in (9), just like phonological rules in the framework of optimality theory. In particular, observers generally prefer to segment a dance movement based on GPR1 (change of moving entity); conversely, they tend not to use GPR6 (change of quality) to place transitions between groups. Further experiments using more various types of movement should determine if the scale in (9) can be confirmed.

**9. Strength scale of grouping preference rules** GPR1 > GPR3 > GPR4 > GPR5 > GPR2 > GPR6.

**Figure 9:**
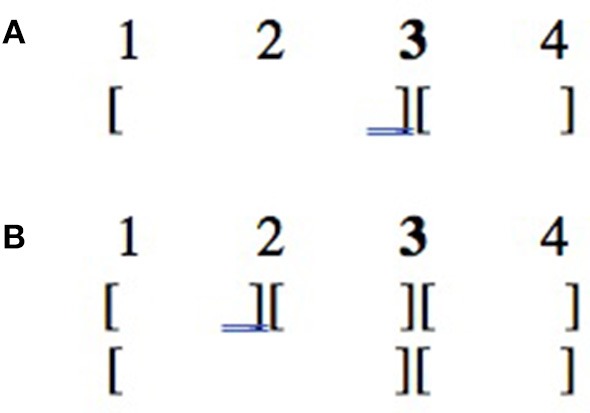
Two possible interpretations of rule strength. 1, 2, 3, and 4, respectively, correspond to the starting position, the point at which the weaker type of change occurs, the point at which the stronger type of change occurs, and the finishing position. **(A)** One-level grouping structure based on the stronger rule of change. **(B)** Two-level grouping structure based on both rules of change at the lower level of representation and on the stronger rule of change at the higher level of representation.

What the experiment leaves unspecified is whether a stronger rule blocks a weaker rule or applies at a higher level of representation. Consider Figure 9 and suppose that a stronger change (e.g., change of body part) occurs at point 3 than at point 2 (e.g., change of orientation). The general results of our experiment are compatible with both representations (a) and (b).

Nevertheless, recall that in most cases, the few participants who did not choose to segment the movement based on the stronger rule, chose to do so based on the weaker rule rather than at any other point. This suggests that representation (b) is more plausible than representation (a), and such participants chose a point of segmentation at the lower level of representation, while most participants were based on the higher one.

Another aspect that was not specifically taken into consideration in the experiment is the difference between categorical change (a change that cannot vary in intensity, e.g., change of body part) and gradual change (a change that can be more or less intense, e.g., change of orientation), and we observe that the only categorical type of change among the 6 tested types of change (i.e., change of body part)^12^ was perceived as the strongest one. This raises the question whether categorical changes are generally perceived as more salient than gradual ones or whether this depends on the intensity of gradual changes.

This question thus relates to the issue of how to precisely compare the intensity of different types of change (e.g., how do orientation changes and speed changes compare? Cf. Deliège (1987) for discussion about a similar issue in music). As explained in section Stimuli, I strove to ensure that the gradual types of change were always performed as highly intensely as possible in the conditions of the experiment to avoid perceived differences of intensity among the different types of change. It is indeed expected that local rules of change interact with the global rule called intensification by Lerdahl and Jackendoff (1983) and adapted as GPR10 in (10).

**10 GPR 10 (intensification)**: When the effects picked out by the local rules of change (GPR1-GPR6) are relatively more pronounced, a larger-level group boundary may be placed.

But the question would be worth further investigating. One way to examine the interaction between intensity and type of change would be to compare pairs of types of change in various intensity conditions (e.g., more or less intense changes of direction compared to more or less intense changes of orientation). An application of this method to various body parts and types of movement should ultimately allow us to establish the relations between the different scales of intensity for each type of change.

## Conclusion

In sum, I hope to have taken a first step toward building a universal syntax of dance: the mental representation of dance movements in observers is governed by grouping principles. On the one hand, this supports the idea that grouping is a general cognitive capacity that applies across different domains and modalities. On the other hand, the dance grouping principles that I have proposed confirm that their realization relies on the specific properties of dance as non-goal-oriented human movement perceived in the visual modality.

Many more steps need to be taken to attain an understanding of the structural principles governing the representation of dance perception^13^. I have here focused on one type of local grouping principle (similarity) marking group boundaries based on six parameters (moving entity, orientation, weight support, direction, speed, quality). More local principles involving other types of principle (such as temporal proximity) and/or parameters could be examined, and global principles such as symmetry or parallelism in dance, which should introduce hierarchy in the system, remain to be examined [see Charnavel (2016) for discussion]^14^. The interaction between these various grouping principles is also to be further understood.

Moreover, grouping is only one dimension of dance organization among others. Lerdahl and Jackendoff (1983) identify other types of structure in music, including in particular metrical structure, time-span reduction (cf. Schenkerian reduction, see Schenker, 1935) and prolongational reduction. We could wonder whether these other types of structure are also relevant in dance (see Charnavel, 2016 for discussion). This would involve investigating whether dance is characterized by its own hierarchical regularity of timing (i.e., a metrical structure independent of music) and if so, how it is realized in the visual modality [cf. studies on music-induced movements such as Toiviainen and Thompson (2010) and Su (2016), i.a.]. This would also require inquiring into other types of hierarchical structure in dance, which could rely on the notion of stability: just like music, dance could be subject to a reductional structure, where the most stable elements are perceived as heads forming the core structure of the movement; dance could also involve nested patterns of tension and relaxation, determined in part by changes in stability.

Finally, it would be worth examining the interaction between dance and music structures (cf. Jordan, 2000; Leaman, 2016, and other choreomusical studies)^15^. This should be informative for specifying not only the structural components of dance itself, but also their relation with the structural components of music, which belongs to the same domain (non-referential art), but to a different modality (auditory vs. visual). Similar comparisons could also be done with other types of cognitive systems, in particular between dance and sign languages, which share the same visual modality, but in a different domain (referential system). In sum, the exploration of an understudied cognitive system, dance, not only for itself but also in comparison with other cognitive systems, promises to shed further light on the underlying organization of our mental representations in further specifying the distinction between domain-specific, modality-specific, and general cognitive properties.

## Ethics Statement

This study was carried out in accordance with the recommendations of the Harvard Committee on the Use of Human Subjects with written informed consent from all subjects. All subjects gave written informed consent in accordance with the Declaration of Helsinki. The protocol was approved by the Harvard Committee on the Use of Human Subjects.

## Author Contributions

The author confirms being the sole contributor of this work and has approved it for publication.

### Conflict of Interest Statement

The author declares that the research was conducted in the absence of any commercial or financial relationships that could be construed as a potential conflict of interest.

## Footnotes

^1^Studies in dance ethnology also adopt linguistic notions to describe various dance systems (see references above, cf. also Myers, 1979; Quale, 1999, i.a.).

^2^That is not to say that the grouping principles that we will examine are not relevant to the artistic and aesthetic aspect of dance. They most probably are, but I do not focus on this question here and leave it for future research.

^3^For simplicity, I will focus on individual movement performances here, but note that the same types of questions arise with group performances. In the latter case, grouping structures of the movement can interact with the grouping structures of the dancers.

^4^We furthermore observe interactions between different domains in some cases. For instance, both prosodic and musical structures seem to be relevant in poetry (see Lerdahl, 2001, i.a.).

^5^Lerdahl and Jackendoff (1983) also use some transformational rules to account for grouping overlap and elision. These cases also arise in dance: the same position (e.g. *plié* in ballet) can serve as the end of a step and the beginning of another step (overlap), and occlusion can occur when a (part of a) dancer hides another (part of a) dancer.

^6^Another principle argued to affect musical local grouping by Jackendoff and Lerdahl (2006) is temporal proximity. It is however unclear whether this principle is also relevant in dance structure given that positions in dance, unlike notes in music, are continuous. The equivalent of temporal proximity can probably be captured by GPR5.

^7^As noted by a reviewer, the similarity to Optimality Theory's predecessor, Harmonic Grammar (Legendre et al., 1990) may be stronger given that in this type of grammar, each constraint can be weighted and several weaker constraints together can beat out a stronger constraint. The application of multiple GPRs is however not tested in this paper, but would be worth testing in future work.

^8^As noted by a reviewer, I here ignore the possible variation in speed of the orientation change (in the rotating movement of the arm). It would be interesting to test whether this could have an effect on the findings.

^9^The study was carried out in accordance with the Declaration of Helsinki (the research was determined as exempt by the Harvard Committee on the Use of Human Subjects).

^10^As mentioned by a reviewer, it would nevertheless have been safer to do an a priori power analysis that justified the sample size. I hope that future replications of this type of study will remedy this potential issue.

^11^The difference of strength between rules may be further supported by the fact that participants who selected the stronger rule had significantly more confidence in their choice than those who selected the weaker rule (see results in the Supplementary Material): a heteroscedastic *t*-test (performed on averages by item) indicates that confidence rates for choices of stronger rules are significantly higher than confidence rates for choices of weaker rules (*p* = 0.026). For instance, the participants who selected the change of body part as the point of segmentation of item 1 reported having a level of confidence of 83.73%, as compared to 74% for those who selected the change of orientation. As mentioned above and noted by a reviewer, these additional results are however only suggestive given that the number of participants in each group significantly varied.

^12^GPR3 involves a categorical change if it is about changing the body part in contact with the floor, but a gradual one if it is about shifting weight.

^13^As specified in the introduction, I am not interested in the artistic aspect of dance here (just like linguists are not interested in the artistic effects of texts). To reach a full understanding of the structure of dance as an artistic object, it would furthermore be necessary to investigate all rules (applied by the choreographer) related to art creation. This is a different topic.

^14^Some of these principles can be instantiated in different ways depending on the modality. As noted by a reviewer, it is for instance much less common to find left-right symmetry in music than in dance.

^15^For instance, the interaction between the grouping structure of dance and the metrical structure of the accompanying music can give rise to different types of perceived movements: movements that have an upbeat character and movements that have an afterbeat character (e.g., movements performed “the accent in” or “the accent out” in ballet).
