# Supplementary material for: Steps Toward a Universal Grammar of Dance: Local Grouping Structure in Basic Human Movement Perception
Source: Front Psychol. 2019 Jun 18;10:1364. doi: 10.3389/fpsyg.2019.01364 (PMC6592219; doi:10.3389/fpsyg.2019.01364)
Supplement: Supplementary file 1 [file Data_Sheet_1.pdf]

Supplementary material description

The supplementary material includes:

- the 15 full video clips used in the experiment as described in Table 1 below (the depicted individual provided a written informed consent for publication of these videos);
- the results about confidence means in Tables 4bis and 6bis below.
  Table 4bis and Table 6bis are similar to Table 4 and Table 6 in the article except that they additionally include the results about the degree of confidence averaged over participants in each case. As mentioned in footnote 10, these results were not included in the text given that the number of participants significantly varied in each case (for instance in item 1, 26 participants chose body part with a confidence mean of 83.73 whilst 2 participants chose orientation with a confidence mean of 74), which makes these results only suggestive.

Table 1. The combinations of 2 possible changes in the 15 stimuli of the experiment.

	**First change (at 1/3 of the movement)**	**Second change (at 2/3 of the movement)**
**Item 1 (Video 1)**	body part (GPR1)	orientation (GPR2)
**Item 2 (Video 2)**	level (GPR3)	body part (GPR1)
**Item 3 (Video 3)**	direction (GPR4)	body part (GPR1)
**Item 4 (Video 4)**	body part (GPR1)	speed (GPR5)
**Item 5 (Video 5)**	body part (GPR1)	quality (GPR6)
**Item 6 (Video 6)**	orientation (GPR2)	level (GPR3)
**Item 7 (Video 7)**	orientation (GPR2)	direction (GPR4)
**Item 8 (Video 8)**	orientation (GPR2)	speed (GPR5)
**Item 9 (Video 9)**	quality (GPR6)	orientation (GPR2)
**Item 10 (Video 10)**	direction (GPR4)	level (GPR3)
**Item 11 (Video 11)**	level (GPR3)	speed (GPR5)
**Item 12 (Video 12)**	level (GPR3)	quality (GPR6)
**Item 13 (Video 13)**	direction (GPR4)	speed (GPR5)
**Item 14 (Video 14)**	direction (GPR4)	quality (GPR6)
**Item 15 (Video 15)**	quality (GPR6)	speed (GPR5)

Table 4 bis. Detailed results by item: number of participants, percentage of participants and average of confidence degree for each answer option.

		Answer options (beat number appearing on the screen)							
	**Item**	**≈ 4**	**≈5**	**≈6**	**≈7**	**≈8**	**≈9**	**≈10**	**≈11**
**Actual points of changes**	**Item 1- body part/ orientation**				--			---------	
**Number of participants**		n/a	0	7	19	2	0	2	0
**Percentage of participants**		n/a	0	23.33	63.33	6.67	0	6.67	0
**Mean of confidence degree**		n/a	n/a	86.57	82.68	80	n/a	74	n/a
**Actual points of changes**	**Item 2- level/ body part**			-------			--		
**Number of participants**		1	0	4	1	11	13	0	n/a
**Percentage of participants**		3.33	0	13.33	3.33	36.67	43.33	0	n/a
**Mean of confidence degree**		22	n/a	55.5	73	77.73	69.85	n/a	n/a
**Actual points of changes**	**Item 3- direction/ body part**		--			--			
**Number of participants**		1	6	0	0	12	11	0	n/a
**Percentage of participants**		3.33	20	0	0	40	36.67	0	n/a
**Mean of confidence degree**		90	58	n/a	n/a	72.92	82.45	n/a	n/a
**Actual points of changes**	**Item 4- body part/ speed**				--			---	
**Number of participants**		n/a	0	2	20	0	3	5	0
**Percentage of participants**		n/a	0	6.67	66.67	0	10	16.67	0
**Mean of confidence degree**		n/a	n/a	60	80.95	n/a	65	76	n/a
**Actual points of changes**	**Item 5- body part/ quality**			--			---		
**Number of participants**		0	2	26	2	0	0	0	n/a
**Percentage of participants**		0	6.67	86.67	6.67	0	0	0	n/a
**Mean of confidence degree**		n/a	85	81.08	76.5	n/a	n/a	n/a	n/a
**Actual points of changes**	**Item 6- orientation /level**				--------			-------	
**Number of participants**		n/a	1	1	11	0	3	14	0
**Percentage of participants**		n/a	3.33	3.33	36.67	0	10	46.67	0
**Mean of confidence degree**		n/a	56	77	71.36	n/a	73.33	74.57	n/a
**Actual points of changes**	**Item 7- orientation /direction**				--------			--	
**Number of participants**		n/a	1	1	4	6	2	15	1
**Percentage of participants**		n/a	3.333	3.333	13.33	20	6.67	50	3.33
**Mean of confidence degree**		n/a	88	91	68.5	61.33	75	68.07	42
**Actual points of changes**	**Item 8 - orientation /speed**			--------			---		
**Number of participants**		0	0	2	11	9	7	1	n/a
**Percentage of participants**		0	0	6.67	36.67	30	23.33	3.33	n/a
**Mean of confidence degree**		n/a	n/a	79	63.09	73.78	77.57	62	n/a
**Actual points of changes**	**Item 9 - quality/ orientation**				---		---------		
**Number of participants**		0	0	0	0	4	23	3	n/a
**Percentage of participants**		0	0	0	0	13.33	76.67	10	n/a
**Mean of confidence degree**		n/a	n/a	n/a	n/a	78	77.65	73.33	n/a
**Actual points of changes**	**Item 10 - direction/ level**		--				-------		
**Number of participants**		1	7	5	1	4	12	0	n/a
**Percentage of participants**		3.33	23.33	16.67	3.33	13.33	40	0	n/a
**Mean of confidence degree**		61	65.43	77.4	65	66	81.83		n/a

**Actual points of changes**	**Item 11- level/ speed**				--------			---	
**Number of participants**		n/a	0	2	14	2	5	7	0
**Percentage of participants**		n/a	0	6.67	46.67	6.67	16.67	23.33	0
**Mean of confidence degree**		n/a	n/a	81.5	58.36	81	71.4	60.71	n/a
**Actual points of changes**	**Item 12 - level/ quality**			--------			---		
**Number of participants**		0	1	19	9	1	0	0	n/a
**Percentage of participants**		0	3.33	63.33	30	3.33	0	0	n/a
**Mean of confidence degree**		n/a	1	80.26	62.89	69	n/a	n/a	n/a
**Actual points of changes**	**Item 13 - direction/ speed**			---			---		
**Number of participants**		0	0	12	3	4	10	1	n/a
**Percentage of participants**		0	0	40	10	13.33	33.33	3.33	n/a
**Mean of confidence degree**		n/a	n/a	65.67	60	59.75	66.6	39	n/a
**Actual points of changes**	**Item 14 - direction/ quality**			--			---		
**Number of participants**		3	4	20	1	0	2	0	n/a
**Percentage of participants**		10	13.33	66.67	3.33	0	6.67	0	n/a
**Mean of confidence degree**		74.33	63	70.6	39	n/a	84	n/a	n/a
**Actual points of changes**	**Item 15 - quality/ speed**			---			---		
**Number of participants**		3	0	1	5	7	13	1	n/a
**Percentage of participants**		10	0	3.33	16.67	23.33	43.33	3.33	n/a
**Mean of confidence degree**		57	n/a	92	49.6	53.57	76.69	49	n/a

Table 6 bis. Relative strength of the six types of changes: for each change in each item, number of participants who chose target, near-target points and both, their degree of confidence in percentage for each case, binomial tests and ranking of each pair of changes.

Item		target	confidence mean	near-target	confidence mean	target +near-target	confidence mean	binomial test	relative strength
1	**body part**	26	83.73	0	n/a	26	83.73	p<0.001	*body part > orientation
	**orientation**	2	74	0	n/a	2	74		
2	**level**	4	55.5	1	73	5	59	p=0.029	*body part > level
	**body part**	24	73.46	0	n/a	24	73.46		
3	**direction**	6	58	1	90	7	62.57	p=0.002	*body part > direction
	**body part**	12	72.92	11	82.45	23	77.48		
4	**body part**	20	80.95	2	60	22	79.05	p<0.001	*body part > speed
	**speed**	8	71.88	0	n/a	8	71.88		
5	**body part**	26	81.08	2	85	28	81.36	p<0.001	*body part > quality
	**quality**	0	n/a	0	n/a	0	n/a		
6	**orientation**	11	71.36	1	77	12	71.83	p=0.097	level > orientation
	**level**	14	74.57	3	73.33	17	74.36		
7	**orientation**	10	64.2	0	n/a	10	64.2	p=0.063	direction > orientation
	**direction**	15	68.07	2	75	17	68.88		
8	**orientation**	13	65.54	0	n/a	13	65.54	p=0.126	speed > orientation
	**speed**	7	77.57	9	73.78	16	75.44		
9	**quality**	0	n/a	0	n/a	0	n/a	p<0.001	*orientation > quality
	**orientation**	23	77.65	7	76	30	77.27		
10	**direction**	7	65.43	5	77.4	12	70.42	p=0.113	level > direction
	**level**	12	81.83	4	66	16	77.88		
11	**level**	16	61.25	0	n/a	16	61.25	p=0.113	level > speed
	**speed**	7	60.71	5	71.4	12	65.17		
12	**level**	19	80.26	10	56.7	29	72.14	p=0.002	*level > quality
	**quality**	0	n/a	1	69	1	69		
13	**direction**	12	65.67	3	60	15	64.53	p=0.144	direction > speed
	**speed**	10	66.6	4	59.75	14	64.64		
14	**direction**	20	70.6	4	63	24	69.33	p=0.002	*direction > quality
	**quality**	2	84	0	n/a	2	84		
15	**quality**	1	92	5	49.6	6	56.67	p=0.003	*speed > quality
	**speed**	13	76.69	7	53.57	20	68.6		
